# Quality Control and Stability Studies with the Monoclonal Antibody, Trastuzumab: Application of 1D- *vs.* 2D-Gel Electrophoresis

**DOI:** 10.3390/ijms15046399

**Published:** 2014-04-15

**Authors:** Dashnor Nebija, Christian R. Noe, Ernst Urban, Bodo Lachmann

**Affiliations:** 1 Department of Pharmaceutical Chemistry, Medical Faculty, Rr. Bulevardi i Deshmoreve, n.n. 10000 Pristina, Kosovo; E-Mail: dashnor.nebija@uni-pr.edu; 2 Department of Pharmaceutical Chemistry, Faculty of Life Sciences, University of Vienna, Althanstrasse 14, 1090 Vienna, Austria; E-Mails: christian.noe@univie.ac.at (C.R.N.); ernst.urban@univie.ac.at (E.U.)

**Keywords:** trastuzumab, 2D-electophoresis, SDS-PAGE, charge heterogeneity, stability

## Abstract

Recombinant monoclonal antibodies (rmAbs) are medicinal products obtained by rDNA technology. Consequently, like other biopharmaceuticals, they require the extensive and rigorous characterization of the quality attributes, such as identity, structural integrity, purity and stability. The aim of this work was to study the suitability of gel electrophoresis for the assessment of charge heterogeneity, post-translational modifications and the stability of the therapeutic, recombinant monoclonal antibody, trastuzumab. One-dimensional, SDS-PAGE, under reducing and non-reducing conditions, and two-dimensional gel electrophoresis were used for the determination of molecular mass (Mr), the isoelectric point (pI), charge-related isoform patterns and the stability of trastuzumab, subjected to stressed degradation and long-term conditions. For the assessment of the influence of glycosylation in the charge heterogeneity pattern of trastuzumab, an enzymatic deglycosylation study has been performed using *N*-glycosidase F and sialidase, whereas carboxypeptidase B was used for the lysine truncation study. Experimental data documented that 1D and 2D gel electrophoresis represent fast and easy methods to evaluate the quality of biological medicinal products. Important stability parameters, such as the protein aggregation, can be assessed, as well.

## 1. Introduction

There is a fundamental difference between traditional drug substances and biopharmaceuticals. In contrast to traditional Active Pharmaceutical Ingredients (APIs), in which the International Nonproprietary Name (INN) as a rule indicates a defined structure, the INN of a biopharmaceutical generally does not define only one specific molecular entity, but stands for a mixture of similar compounds. These mixtures are mainly a result of differences in the epigenetic modification of a given protein sequence. Due to the fact that biotechnological production results easily in changes of the pattern of these modifications as a result of minor changes of the production conditions, even batch-to-batch variation in the composition of the product of a specific manufacturer has to be expected. It is not surprising that with the advent of the biosimilars of several important biopharmaceuticals, the quality assessment of these drugs has attained additional importance, in particular in view of pharmacovigilance. Changes in the epigenetic modification of a protein result in different molecular entities, which may have an impact on drug safety, due to the potential for adverse immunological reactions. Therefore, strict observation of the quality is of particular importance in this class of compounds. However, it has to be kept in mind that also drug efficacy may be subject to variations or even loss, due to changes in the epigenetic processing of a specific protein. Therapeutic failures may also depend on this aspect.

For many years, our group has been active in the field of the quality assessment of recombinant drugs, in particular by applying the method of 2D-gel electrophoresis, starting with the analysis of Epo [1]. 2D-gel electrophoresis is particularly well suited for this purpose, due to the option for direct visual pattern recognition and the option for easy additional analysis. Recently, we investigated this method for the analysis of monoclonal antibodies trastuzumab and rituximab [2]. In the present paper, we report our follow-up work on trastuzumab, particular in view of the suitability of the method for stability studies.

Trastuzumab (FDA, USAN INN; Herceptin (trade name); synonyms: huMAb 4D5-8; HER2 receptor monoclonal antibody, recombinant; anti-p185, rhuMab HER2; c-erbB2 monoclonal antibody. Drug Bank ID: DB00072; CAS-180288-69-1) is a recombinant, DNA-derived, humanized monoclonal antibody glycoprotein that selectively targets the extracellular domain of the human epidermal growth factor receptor 2 protein (HER2). The antibody is an IgG1 kappa produced in recombinant Chinese hamster ovary cells and contains human framework regions with the complementarity-determining regions of a murine anti-p185HER2 antibody that binds to HER2 [3,4]. Trastuzumab is composed of 1328 amino acids. It contains two identical heavy (HC) and two identical light chains (LC) linked via disulfide bonds [2,5]. Each HC contains 450 amino acids, and each LC contains 214 amino acids. The respective theoretical molecular masses (Mr) and pI values of HC and LC are 49,284.65 Da and 8.49 for HC and 23,443.1 Da and 7.76 for LC. The theoretical Mr of pI nonglycosylated trastuzumab is 145,455.5 Da [6,7]; however, the apparent Mr of trastuzumab is higher (~148 kDa), due to the presence of *N*-linked oligosaccharides [8]. Like other IgG1 molecules, trastuzumab has one *N*-linked biantennary oligosaccharide on the conserved asparagine residue at position 300, buried between the CH2 domains [5]. The pharmaceutical formulation of trastuzumab (Herceptin^®^, Roche) is a sterile, white to pale yellow, preservative-free lyophilized powder for intravenous infusion. In the EU, trastuzumab is marketed as single-dose formulation (each vial contains 150 mg trastuzumab), whereas in the USA, it is approved as a multi-dose formulation (each multi-use vial of Herceptin^®^ contains 440 mg of active substance). It is indicated for the treatment of HER2 overexpressing breast cancer, metastatic gastric or gastro esophageal junction adenocarcinoma [9,10]. Numerous modern analytical techniques and multiple complementary assays have been used for the quality evaluation of trastuzumab. Biological methods, including-antibody dependent cellular cytotoxicity and the antiproliferation activity potency test, were used for the identification and potency determination of trastuzumab. The latter test is able to indicate the loss of biological activity under conditions of thermal, mechanical, light exposure, oxidative and pH stress and was used as a lot release potency test [11,12]. In addition, for the identity and potency evaluation of trastuzumab, peptide mapping, isoelectric focusing (IEF), capillary isoelectric focusing (cIEF), SDS-PAGE and SDS-capillary gel electrophoresis (SDS-cGE) are used, as well [13–15]. High-performance liquid chromatography (HPLC) coupled with Electrospray ionization-Mass spectrometry (ESI-MS) was used for the quantitation of intact trastuzumab [16], and the enzyme linked immune sorbent assay (ELISA) is used for the determination of trastuzumab serum levels [17,18]. Glycosylation and protein modifications are important, not only for drug stability and efficacy, but also they may have consequences for the patient; for example, incorrect modifications, including aggregation may lead to an immune response to the therapeutic monoclonal antibody [19]. Capillary electrophoresis-laser-induced fluorescence (CE-LIF), CE-ESI-MS, HPLC-ESI-MS, Ultra Performance Liquid Chromatography-Fluorescence Detection (UPLC-FLR)-MS are used for the determination of the glycan profiling of trastuzumab. These methods were able to detect typical, core fucosylated, asialo-biantennary complex-type oligosaccharides (major components) and the sialo-biantennary complex-type and high mannose-type oligosaccharides (minor components) [14,20–25].

Cation exchange chromatography was used for the study of the trastuzumab charge heterogeneity [5,26]. The impact of lyoprotectants on the stability of trastuzumab was reported [27]. The stability of ready-to use trastuzumab infusion solutions was studied by size-exclusion HPLC, UV-Vis spectrometry and SDS-PAGE [28]. The influence of process shear stressors on the stability trastuzumab was studied using SDS-PAGE, size exclusion chromatography (SEC) and circular dichroism (CD) [29]. The aggregation state of trastuzumab in different solutions was characterized by UV-Vis absorbance, asymmetrical flow field-flow fractionation (FFF), fluorescence microscopy, transmission electron microscopy (TEM), 90° light scatter, Nile red fluorescence microscopy, light microscopy, 90° light-scattering and surface plasmon resonance (SPR) [30–33].

Along with other physicochemical, biochemical and immunochemical analytical methodologies, gel electrophoresis has been used for the characterization of the quality of biopharmaceuticals. The advantages of 2D-gel electrophoresis, including its robustness and the capability to analyze complete proteins with a high resolving power, make this method suitable for the analysis of complex protein drugs, such are rmAbs. For the study of chemical modifications (including co- and post-translational modifications), 2D-gel electrophoresis can be easily interfaced with other biochemical (e.g., antibody based) and physico-chemical methods, such is mass spectrometry. Furthermore, peptide mass finger printing by Matrix-assisted laser desorption/ionization (MALDI-TOF)-MS can be used for the identification of biopharmaceuticals following their separation by 2D-gel electrophoresis [34–36].

Monoclonal antibodies are prone to a variety of physical and chemical degradation pathways. Like other proteins, they are particularly susceptible to temperature changes, oxidation, light, ionic content and shear. These factors can affect their potency, purity and quality [37]. Two major pathways of physical instability are denaturation and degradation. Precipitation and surface adsorption occur, as well. Chemical degradation pathways include cross-linking, deamidation, isomerization, oxidation and fragmentation [38].

Regulatory guidelines specify that the expiration dating of biopharmaceuticals should be based on real-time/real-temperature data. However, it is suggested that the stability studies should be conducted under accelerated and stress conditions. These conditions should mimic what could be experienced during drug distribution/transportation or due to inappropriate product handling. Accelerated and stress testing are also useful for evaluating which analytical methods are best for determining product stability and evaluating which specific test parameters may be the best indicators of product stability. Studies of the exposure of the drug substance or drug product to extreme conditions may help to elucidate patterns of degradation. These studies should address the effects of temperature, humidity, oxidation, photolysis and hydrolysis across a wide range of pH values [37–39].

## 2. Results and Discussion

### 2.1. Charge Heterogeneity Study

**Figure 1.**
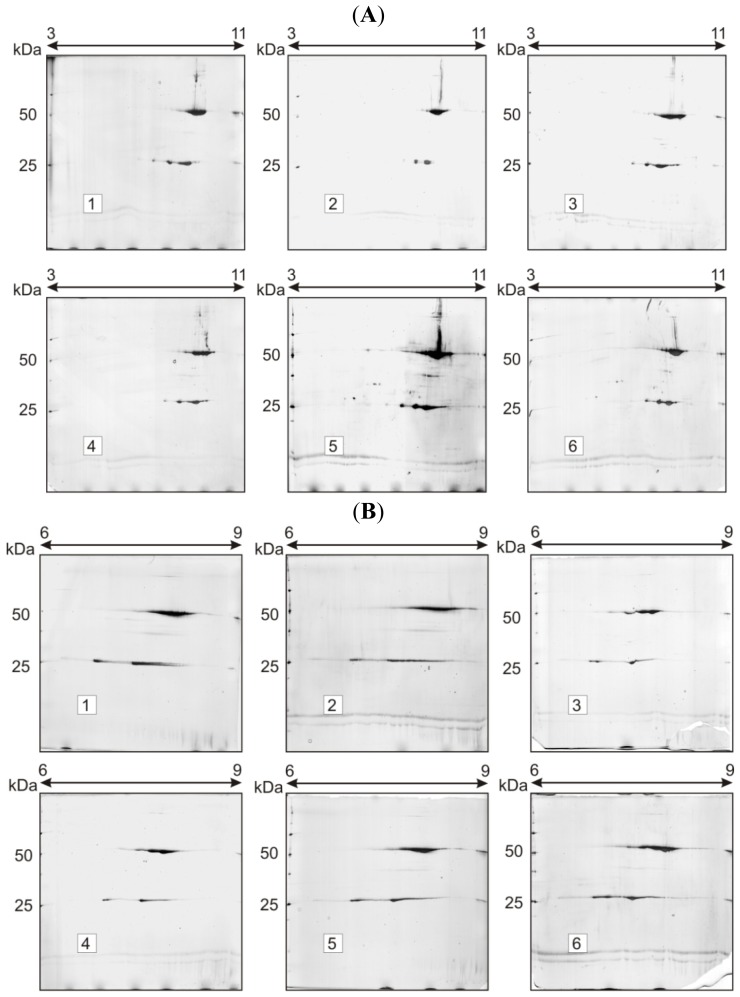
Charge heterogeneity study of trastuzumab (10 μg). Method: Two-dimensional gel electrophoresis (2-DE) with silver staining: (**A**) immobilized pH gradients (IPG) 3–11 nonlinear (NL) and (**B**) IPG 6–9 L. Samples: 1 = control (no enzymatic treatment); 2 = sample treated with sialidase; 3 = sample treated with carboxypeptidase B, sialidase and *N*-glycosidase F; 4 = sample treated with *N*-glycosidase F; 5 = sample treated with carboxypeptidase B; 6 = sample treated with carboxypeptidase B and sialidase. Under both gradient conditions, enzymatic deglycosylation, desialination and carboxypeptidase B cleavage brought about no significant changes in the Mr, pI and spot complexity of the treated samples, compared to control gels.

SDS-PAGE showed that under reducing conditions, trastuzumab migrated as two sharp bands at ~50 and ~23 kDa, corresponding to Mr of the respective HC (heavy chain) and LC (light chain) polypeptides, whereas under non-reducing conditions, a single band was observed at ~150 kDa (intact antibody). In accordance with previously published data, 2D-electrophoresis showed multiple spots spreading at ~50 and ~23 kDa; numerous poorly resolved spots were observed in both chains, particularly in HC, the most intense spots laying on the basic end [2] (Figure 1). In order to assess the contribution of *N*-linked glycans and the *C*-terminal lysine truncation in the spot complexity of trastuzumab, three selected enzymes have been employed: *N*-glycosidase F, sialidase and carboxypeptidase B.

Two-dimensional gel electrophoresis (2-DE) experiments involving *N*-deglycosylation, desialination and terminal lysine cleavage demonstrated no significant changes in the pIs and Mr of trastuzumab HC and LC, as shown in Figure 1 (Immobilized pH gradient, IPG 3–11, nonlinear, NL and IPG 6–9 linear, L, respectively). As we reported previously [2], the high value of gel-to-gel variation in the spot intensity does not allow the detection of these small changes. These data were expected, since in the trastuzumab molecule, *N*-linked, asialo, biantennary complex oligosaccharides are dominant and, according to the literature data, only a minor percentage of terminal sialidated sugars is present (1%–3%) [8,40]. Furthermore, HC terminal lysine truncation can be a source of charge heterogeneity; however, it can bring about only small pI difference between the “normal” and “truncated” form of the antibody, and this difference could not be detected with 2D-electrophoresis.

### 2.2. Stability Study

**Figure 2.**
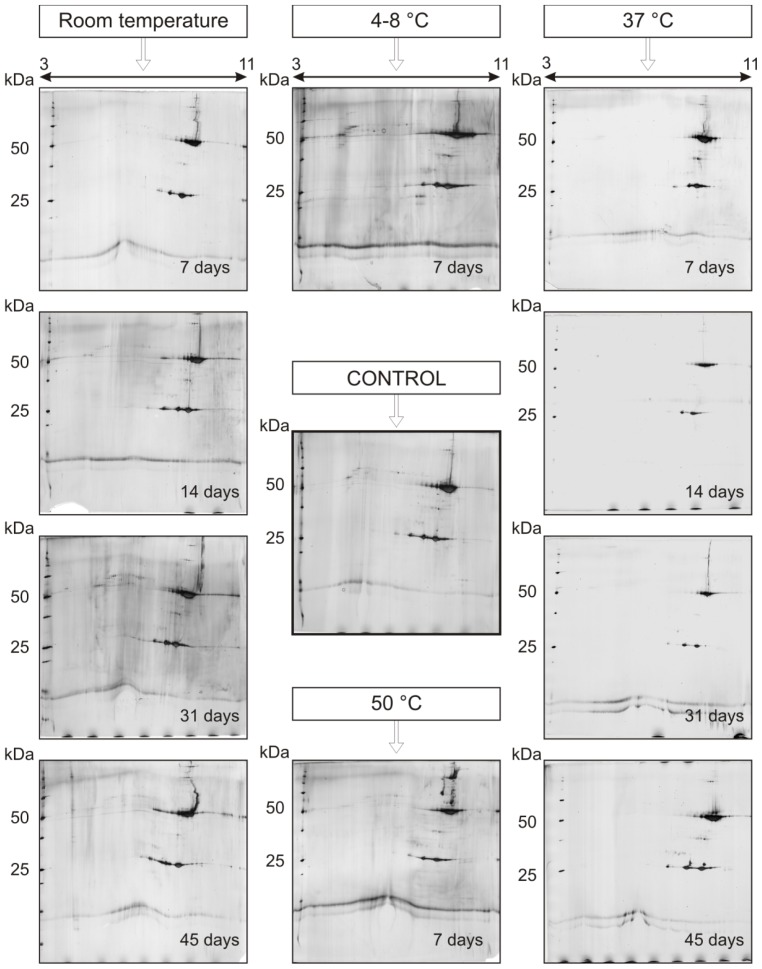
The temperature dependence of trastuzumab stability upon storage for 7, 14, 31 and 45 days. Method: 2-DE (3–11 NL) with silver staining. Trastuzumab, 10 μg. Storage conditions: gels on the left, ambient temperature; gels on the right, 37 °C: Control, 4–8 and 50 °C (in the center of the figure). Compared to the control gel, the change in the spot pattern can be noted only after longer storage times (*i.e.*, 31 and 45 days), particularly in samples subjected to elevated temperatures. Numerous higher molecular spots have been detected in samples stored at 50 °C.

**Figure 3.**
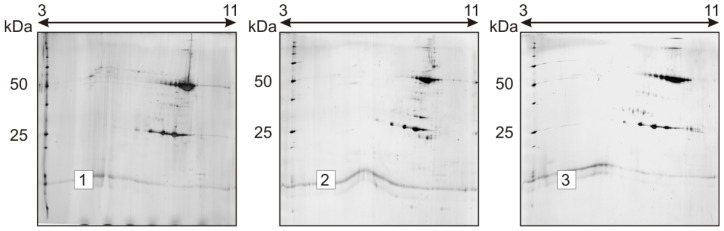
Short-term stability of trastuzumab at ambient temperature: oxidative and pH dependent changes. Method: 2-DE (3–11 NL) with silver staining. Samples: 1 = control; 2 = acidic medium (trifluoroacetic acid, TFA 1%) for seven days; 3 = oxidative medium (H_2_O_2_ 3%) for seven days.

This study addressed the thermal degradation of trastuzumab under accelerated storage conditions. 1-DE and 2-DE analyses were evaluated for their ability to monitor possible changes in the electrophoretic pattern of trastuzumab upon forced degradation conditions, to detect possible degradation products and to assess the effect of other severe conditions (temperature, oxidation and low pH) in the stability of this drug product. In Figure 2, a thermo-degradation study of mAb, trastuzumab solution stored at different temperature conditions is presented. 2-DE analysis showed that, in accordance with published data [29], trastuzumab, like other antibodies, may be a protein resistant to short exposure to thermal and physical stress. As depicted in Figure 2, the influence of temperature in the spot pattern of the antibody is remarkable only upon longer time storage. Storage for 30 and 45 days at room temperature resulted in a change of the shape of both HC and LC spot patterns. In samples stored 45 days at 37 °C, new spots located above LC and HC are noted, and in those stored seven days at 50 °C, new spots can be seen at pI 7.5–8 at Mr ~55, ~75 and ~100 kDa, suggesting the possible aggregation of the antibody molecule (Figure 2). On the other hand, no significant difference in the spot pattern has been noted in gels stored for a shorter time, even at 37 °C. Neither the Mr nor pI of spots changed significantly and consistently. This suggests that the degradation pathway of trastuzumab could involve chemical reactions, such as asparagine deamidation, asparagine and aspartate isomerization and methionine oxidation, which does not cause bigger differences in Mr and pI of the resulting species to be detected with 2-DE. Similar results were obtained when electrophoresis experiments were run under a linear pH gradient, IPG 6–9 L (data not presented). On the other hand, significant differences in the pI and Mr of trastuzumab 2-DE spots are observed upon seven-day sample storage in a solution of 1% trifluoroacetic acid, (Figure 3, Gel 2) and 3% hydrogen peroxide solution (Figure 3, Gel 3), at ambient temperature. Under these conditions, significant changes were observed, both in the pI and Mr of the resulting spots, provided that elevated temperatures substantially accelerated degradation, eventually leading to the complete degradation and denaturation of the sample (data not shown).

**Figure 4.**
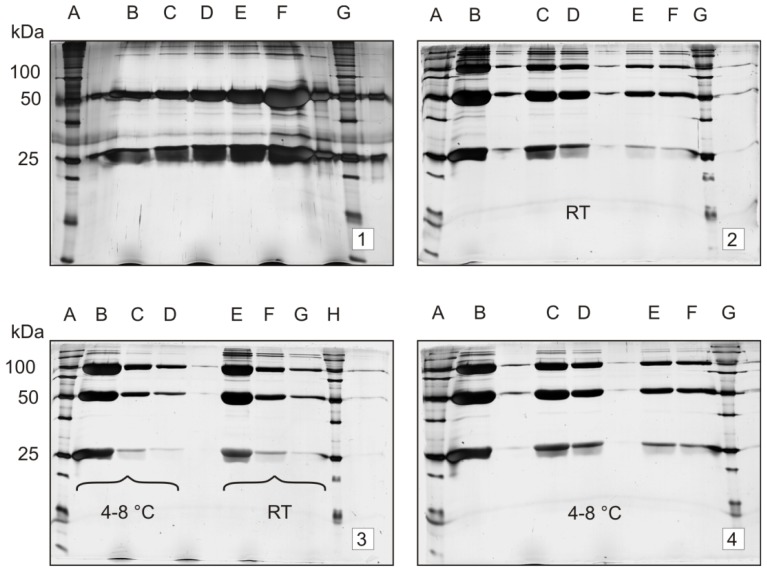
Temperature dependence of trastuzumab stability upon storage for 31 days; the conditions include refrigerated and ambient temperature. Method: 1D-electrophoresis with silver staining; trastuzumab, 1.3–20.5 μg. Molecular weight standards on Lanes A and G for Gels 1, 2 and 4; on Lanes A and H for Gel 3. Gel 1 = control gel (no storage): trastuzumab, 1.3–20.5 μg on Lanes B–F; Gel 2 = samples stored at ambient temperature: trastuzumab 20.5–1.3 μg on Lanes B–F; Gel 3 = samples stored at 4–8 °C *vs.* ambient temperature: trastuzumab 20.5–1.3 μg on Lanes B–D for refrigerated samples and E–G for ambient temperature; Gel 4 = samples stored at 4–8 °C. Trastuzumab, 20.5–1.3 μg on Lanes B–F.

Although, from SDS-PAGE gels, no information regarding pI can be obtained, the Mr changes of trastuzumab under different storage conditions in this electrophoresis setting are evident. Samples stored at room temperature upon 31 days provided gels with additional bands at higher Mr (Figure 4, Gels 2, 3 and 4). In contrast to the control sample (Figure 4, Gel 1), in Gel 2, the trastuzumab sample was stored 31 days at ambient temperature; there is a sharp band at a molecular mass of 100 kDa, which has almost the same intensity with heavy chain band (50 kDa). Additional bands are present at molecular masses of 150 and 200 kDa, respectively.

Figure 4 shows that a higher number of protein bands is observed when samples were stored at ambient temperature (Gels 2 and 3, Lanes E, F and G and Lanes B, C, D, E, F, respectively) compared to samples stored at 4–8 °C (Gels 4 and 3, Lanes B, C and D). This observation may suggest trastuzumab aggregation as a physical degradation pathway.

A different lot of the trastuzumab sample was loaded onto Gel 3.

**Figure 5.**
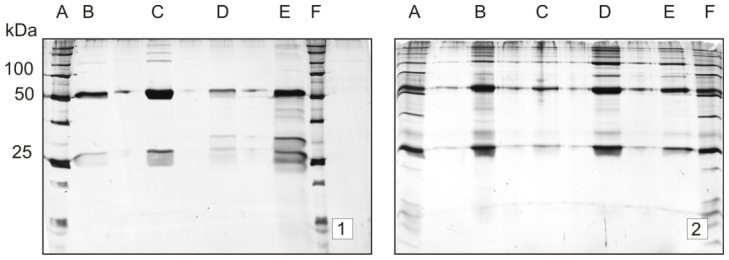
Stability of trastuzumab upon storage for 19 days: temperature-, oxidative- and pH-dependent changes. Method: 1D-electrophoresis with silver staining; trastuzumab: 0.2–0.8 μg. Molecular weight standards on Lanes A and F. Gel 1 = samples stored at 37 °C, Lanes B, C; acidic medium (TFA 1%) for 19 days at ambient temperature; Gel 2 = oxidative medium, H_2_O_2_, 3%, for seven days at ambient temperature, Lanes B, C; H_2_O_2_, 1%, for seven days at ambient temperature, Lanes D, E.

SDS-PAGE gels of trastuzumab stored under oxidative and acidic conditions are presented in Figure 5. As noted in Figure 5, severe storage conditions, employing Trifluoroacetic acid (TFA) 1% (Gel 1, Lanes D and E) and H_2_O_2_ 1 and 3%, 19 days, (Gel 2, Lanes D, E and B, C, respectively), brought about a significant change in the protein band pattern compared to the control gel (Figure 4, Gel 1). Interestingly, additional protein bands above and below 50 kDa have been observed in H_2_O_2_ subjected samples. However, samples stored in the solution of TFA 1% provided additional bands predominantly below 50 kDa (Gel 1, Lanes d and E). On the other hand, samples stored 19 days at 37 °C provided additional bands above 50 kDa, similarly as in Figure 4, (Gel 2).

The left part of this gel (Lanes B, C and D, trastuzumab stored at 4–8 °C, 31 days) shows the same Mr pattern as Gel 4. The same holds for Lanes E, F, G and Gel 2 (ambient temperature, 31 days).

## 3. Experimental Section

### 3.1. Isoelectric Focusing (IEF)

Just prior to use, 1-mL aliquot of rehydration solution (urea, 8 M; thiourea, 2 M; 3-[(3-Cholamidopropyl)dimethylammonio]-1-propanesulfonate (CHAPS) 4%; Triton-X100 0.5%; bromophenol blue 0.005%) was carefully thawed and mixed with 5 mg/mL of dithiothreitol (DTT) and 5 μL/mL IPG buffer of selected pH interval (GE Healthcare Bio-Sciences AB, Uppsala, Sweden). Three hundred and forty microliters of this solution were mixed with 10 μL of the sample solution (1–5 μg/μL trastuzumab) and vortexed briefly. The solution was incubated for 1 h at room temperature and centrifuged (15,000× *g*, 5 min, 20 °C). Strip Holders were put onto the cooling plate/electrode contact area of the IPGphor strip holder platform (IPGPhorTM IEF system, GE Healthcare, Biosciences AB, Uppsala, Sweden). The prepared sample solutions were applied onto Immobiline DryStrip gels (6–9 L and 3–11 NL, 18 cm ± 2 mm, GE Healthcare Bio-Sciences AB, Uppsala, Sweden) by in-gel rehydration according to [41] and focused for a total of 72 kVh.

### 3.2. Second Dimension SDS-PAGE

Before loading onto SDS-polyacrylamide gels, focused IPG strips were equilibrated twice, by gently shaking for 15 min in SDS equilibration buffer (50 mM Tris–HCl, 6 M urea, 30% glycerol, 2% SDS, 0.005% bromophenol blue) containing 1% DTT and then for another 15 min in SDS equilibration buffer containing 2.5% iodoacetamide [42]. The equilibrated IPG strips were embedded on top of the vertical SDS gel and fixed with molten agarose solution (Agarose Serva Standard low EEO, research grade (Serva Electrophoresis GmbH, Heidelberg, Germany)). The second-dimension, SDS-PAGE, was run on a vertical system (PROTEAN^®^ II Xi Cell for vertical electrophoresis, 20 cm × 20 cm, lab-made gels 12.5% T (total amount of acrylamide), homogenous, 2.6% C (amount of cross-linker), Bio-Rad Laboratories, (Hercules, CA, USA) under a constant current of 45 mA/gel until the bromophenol blue front reached the bottom of the gel. Molecular mass standards (Precision Plus Unstained Protein™ Standards, Bio-Rad Laboratories) were placed at the anodic end of the SDS-PAGE gel. One-dimensional, SDS-PAGE was performed on the mini-gel system, Mini-PROTEAN^®^ 3 Cell (Bio-Rad Laboratories), according to standard methods [43]. Silver nitrate staining was performed as described in [44], whereas gel images were digitized using a Bio-Rad GS-710 Densitometer and analyzed with PDQuest 6.2.1. (Bio-Rad Laboratories).

Enzymatic deglycosylation of trastuzumab was carried out according to the published procedures [2,8]. Solutions of the pharmaceutical preparation, trastuzumab (Herceptin^®^, F. Hoffmann-La Roche, Basel, Switzerland) 21 mg/mL collected from the vials immediately after clinical use in Vienna Hospital Rudolfstiftung, were dialyzed against Milli-Q water (3 days, 4 °C) using a cellulose dialysis tube (cut-off: 12,000–14,000 Da, Japan Medical Science, Tokyo, Japan). After dialysis, solutions were lyophilized, and samples of pharmaceutical preparations, 0.5 mg each, were dissolved in 49 μL phosphate buffer (pH 7.5) to obtain a final concentration of 500 μg/50 μL. *N*-Glycosidase F, recombinant, from *Flavobacterium meningosepticum*, expressed in *E. coli*, 100 U/100 μL was used for the release of *N*-linked oligosaccharides, (15 U/150 μg rmAb). Neuraminidase (sialidase) from *Arthrobacter ureafaciens*, 100 U/0.1 mL, 30 mU/150 μL rmAb, was used for the removal of sialic acids. The study of the lysine truncation was performed using enzyme carboxypeptidase B, 750 U/mL, (0.75 U/150 μg rmAb). All enzymes were purchased from Roche Diagnostics, Mannheim, Germany. After overnight digestion at 37 °C, samples were stored at −80 °C.

The rate of chemical degradation was increased by subjecting the reconstituted pharmaceutical formulation, Herceptin^®^, to elevated temperatures, oxidation (hydrogen peroxide solution, 1% and 3%) and low pH conditions (trifluoroacetic acid, 1%). For the thermal stability study, the drug substance was stored at ambient temperature, 4–8, 37 and 50 °C in a Thermomixer comfort (Eppendorf AG, Hamburg, Germany), and after storage up to a given time, the sample was analyzed with 1D- and 2D-electrophoresis. The testing frequency was weekly for 2 months and daily for 1 week.

## 4. Conclusions

This study confirms that SDS-PAGE and 2D electrophoresis can be used for the quality evaluation of recombinant monoclonal antibodies. These easy and simple techniques can be used for the identification, assessment of purity and aggregation of biological medicinal products. Compared to SDS-PAGE, in relation to the charge heterogeneity pattern of antibodies, 2-DE has been shown to be a superior method. However, experimental results obtained from the stability study suggested that SDS-PAGE can be more suitable for the detection of higher molecular mass species, *i.e.*, aggregates, which can arise upon different storage conditions of this monoclonal antibody. On the other hand, for the comprehensive stability characterization of complex drugs, including recombinant mAbs, 1D-/2D-electrophoresis alone can provide limited information. Therefore, for this purpose, a combination of different analytical methodologies is required.
